# The cost of a primary care-based childhood obesity prevention intervention

**DOI:** 10.1186/1472-6963-14-44

**Published:** 2014-01-29

**Authors:** Davene R Wright, Elsie M Taveras, Matthew W Gillman, Christine M Horan, Katherine H Hohman, Steven L Gortmaker, Lisa A Prosser

**Affiliations:** 1 Department of Pediatrics, University of Washington School of Medicine, PO Box 5371, M/S: CW-8-6, 98145-5005 Seattle, Washington, USA; 2 Division of General Academic Pediatrics, Department of Pediatrics, Massachusetts General Hospital for Children, Boston, Massachusetts, USA; 3 Obesity Prevention Program, Department of Population Medicine, Harvard Medical School and Harvard Pilgrim Health Care Institute, Boston, Massachusetts, USA; 4 Department of Nutrition, Harvard School of Public Health, Boston, Massachusetts, USA; 5 Healthy Living Department, YMCA of the USA, Chicago, Illinois, USA; 6 Department of Society, Human Development and Health, Harvard School of Public Health, Boston, Massachusetts, USA; 7 Child Health Evaluation and Research Unit, Division of General Pediatrics, University of Michigan Health System, Ann Arbor, Michigan, USA

**Keywords:** Child, Preschool, Obesity, Cost, Economic evaluation

## Abstract

**Background:**

United States pediatric guidelines recommend that childhood obesity counseling be conducted in the primary care setting. Primary care-based interventions can be effective in improving health behaviors, but also costly. The purpose of this study was to evaluate the cost of a primary care-based obesity prevention intervention targeting children between the ages of two and six years who are at elevated risk for obesity, measured against usual care.

**Methods:**

High Five for Kids was a cluster-randomized controlled clinical trial that aimed to modify children’s nutrition and TV viewing habits through a motivational interviewing intervention. We assessed visit-related costs from a societal perspective, including provider-incurred direct medical costs, provider-incurred equipment costs, parent time costs and parent out-of-pocket costs, in 2011 dollars for the intervention (n = 253) and usual care (n = 192) groups. We conducted a net cost analysis using both societal and health plan costing perspectives and conducted one-way sensitivity and uncertainty analyses on results.

**Results:**

The total costs for the intervention group and usual care groups in the first year of the intervention were $65,643 (95% CI [$64,522, $66,842]) and $12,192 (95% CI [$11,393, $13,174]). The mean costs for the intervention and usual care groups were $259 (95% CI [$255, $264]) and $63 (95% CI [$59, $69]) per child, respectively, for a incremental difference of $196 (95% CI [$191, $202]) per child. Children in the intervention group attended a mean of 2.4 of a possible 4 in-person visits and received 0.45 of a possible 2 counseling phone calls. Provider-incurred costs were the primary driver of cost estimates in sensitivity analyses.

**Conclusions:**

High Five for Kids was a resource-intensive intervention. Further studies are needed to assess the cost-effectiveness of the intervention relative to other pediatric obesity interventions.

**Trial registration:**

ClinicalTrials.gov Identifier: NCT00377767.

## Background

In response to the growing prevalence of childhood obesity, the 2010 Patient Protection and Affordable Care Act promotes the utilization of preventive care, mandating that insurers cover obesity screening and counseling services as part of insurance policies [1]. In 2007, a U.S. expert committee recommended that obesity counseling be conducted in the primary care setting, specifically suggesting that pediatricians use patient-centered techniques such as motivational interviewing to counsel patients about improving health behaviors [2]. The use of motivational interviewing techniques in clinical settings has been reported to be programmatically feasible and effective in improving health behaviors [3,4], but it is also important to assess the time and financial resources needed to implement such programs. Decision makers such as health providers, health care payers, and policymakers can use analyses of the costs of health interventions to assess the affordability of interventions and to prioritize resources among competing programs.

Only a dozen analyses in the peer-reviewed literature detail the costs of delivering childhood obesity interventions [5-16]. Just one study, the Australian Live, Eat, and Play (LEAP) intervention, has supported evaluations of the cost of a primary care-based obesity intervention trial [9-11]. The cost of LEAP varies between studies, so we cannot draw definitive conclusions about the affordability of a primary care-based obesity prevention program from those analyses. No analyses have evaluated the cost of primary care-based obesity counseling in the context of the U.S. health care system.

High Five for Kids was an obesity prevention program developed for 2-6.9 year old children who were obese, or overweight with at least one overweight parent [4]. The intervention has been found to be effective in decreasing television viewing and decreasing unhealthy eating practices among young children [4]. The aim of this study was to assess the costs associated with implementing the High Five for Kids intervention measured against usual care practices.

## Methods

### High Five for Kids intervention

High Five for Kids was a cluster-randomized controlled trial conducted from 2006-2008 in 10 non-profit private pediatric offices of Harvard Vanguard Medical Associates, a multi-site group practice in eastern Massachusetts. There were 5 intervention groups (n = 253 children) and 5 usual care practice groups (n = 192 children). The intervention was based on the Chronic Care Model, which suggests that all members of the pediatric care practice team should be actively involved in an intervention in order to successfully change patient behaviors and improve health outcomes [17]. As such, the electronic medical record system was enhanced to assist clinicians with decision support and in tracking patient progress, and all members of the health care practice team, from the receptionist to the physician, received training on obesity management guidelines. The intervention consisted of four extended “chronic disease management” visits and two telephone calls with parents of children conducted by trained nurse practitioners (Advanced Practice Clinicians, or APCs). Study investigators negotiated with health plans to pay for these visits for overweight and obese patients, including reimbursing parents for any visit-related co-payments; all participants were privately insured. APCs used motivational interviewing techniques to help parents identify problematic unhealthy behavioral practices related to television watching and nutrition. As part of the intervention, the research team developed materials, including posters and an interactive website with recipes and educational literature, to assist the practices in supporting behavior changes in families. The APCs provided participants with incentives such as water bottles and books, and electronic television monitoring devices to facilitate reducing television viewing time. Detailed methods of the trial are reported elsewhere [18]. All study procedures were approved by the Human Subjects Committee of Harvard Pilgrim Health Care.

### Measuring intervention costs

We assessed costs of the motivational interviewing intervention, including the cost of study-related visits incurred by the provider, costs incurred by the parents of children while attending study visits, intervention training costs, and the cost of materials and equipment for the intervention and usual care groups. The inclusion of costs incurred by the parents of children in the trial allowed us to conduct our analysis from the societal perspective, considering the investment of time and financial resources made by every person involved in the intervention [19]. Costs were adjusted to 2011 US dollars using the medical component of the Consumer Price Index [20]. Our primary outcome was the net cost of the intervention, that is, the difference in costs for the intervention group compared to the usual care group.

Provider-incurred costs included the cost of study-related visits, materials, and equipment. We did not measure costs related to healthcare utilization of non-study-related visits. In the base case, we calculated the cost of study-related visits and phone counseling using a micro-costing approach by multiplying APC wage data by the time APCs spent counseling parents in person and by phone. We derived overhead costs using published estimates of overhead costs per visit in a pediatric practice [21] and data from the trial on the number of in-person visits attended per child. Materials and equipment included intake forms, newsletters, printed handouts, toys, scales, and stadiometers. Scales and stadiometers were necessary for accurate and precise measurement of height and weight between sites.

We administered a survey at the end of year one of the study to estimate parent non-medical and time costs. Parents reported their time, time required for childcare for other children, transportation, and out-of-pocket costs associated with study visits. Parent time costs were valued using the 2007 mean adult US wage rate [22,23]. Childcare time costs were valued using the average household productivity wage rate [19,24].

### Uncertainty analyses

Using a percentile method approach, we conducted uncertainty analyses to assess variability in results from potential sampling bias [25]. We used the mean outcomes from 1,000 bootstrapped samples from the trial data to generate a range of cost outcomes. These results were used to construct confidence intervals around cost estimates as a measure of uncertainty around results.

#### Sensitivity analyses

Sensitivity analyses on alternative costing approaches were conducted to test the robustness of results. First, we considered an approach that included training costs in total intervention costs. Training costs are not conventionally considered in base case analyses, rather, they are typically treated as fixed costs. Secondly, we considered an approach that used health plan reimbursement data to calculate the costs of study visits from a payer perspective as opposed to the micro-costing approach used in the base case. Lastly, we considered a costing approach that annuitized costs for durable equipment that could be used by practices after the completion of the study.

### Training costs

We calculated training costs for intervention site clinical staff and APCs. As the key intervening clinicians, APCs participated in eight 1 to 3 hour-long on-site training sessions conducted by experienced APCs. Intervention site clinical staff (pediatricians, registered nurses, nurse practitioners, and medical assistants) participated in one 1-hour training session, and medical assistants in both study arms attended an additional 1-hour session to learn how to use scales and stadiometers. We obtained wage data for APCs from primary study data. Intervention site clinical staff hourly wages were estimated from the 2007 Bureau of Labor Statistics survey [22].

### Health plan perspective costs

As an alternative to the micro-costing approach employed in the base case, we used reimbursement data obtained from the health plan to calculate the cost of study visits from the health plan perspective. While all intervention participants attended study visits for counseling, reimbursement data were available for only 238 out of the estimated 504 visits because participants did not submit invoices for reimbursement of visit co-pays. We assumed visit cost data was missing at random. We imputed missing visit costs as the mean non-missing visit cost of all children in the trial using data on the number of study visits each child attended.

### Equipment costs

Our base case analysis took a conservative approach to estimating equipment costs, allocating the entire costs of scales and stadiometers to the intervention. However, these equipment costs could be considered capital equipment, with a life expectancy beyond the two-year study time period. In a sensitivity analysis, we considered a more liberal costing approach in which costs were annuitized over the useful life of the equipment. We assumed that scales and stadiometers could both be used for 10 years before being replaced.

## Results

### Costs

The total costs for the intervention group were $65,643 (95% CI [$64,522, $66,842]) or $259 (95% CI [$255, $264]) per child. The total costs for the usual care group were $12,192 (95% CI [$11,393, $13,174]), or $63 (95% CI [$59, $69]) per child (Tables 1 and 2). Intervention participants attended a mean of 2.4 of a possible 4 in-person visits and received 0.45 of a possible 2 counseling phone calls. The number of contacts varied among participants. 56% of participants completed exactly two of the six planned contacts (visits and phone calls) and 15% completed all six contacts. 13 of the 192 subjects in the usual care arm mistakenly received 1 to 7 study-related weight counseling visits each; usual care participants attended a mean of 0.13 in person visits and had no counseling phone calls. Almost 90% of the total intervention group trial costs were comprised of provider-incurred costs. Parents spent a mean of 65.2 minutes attending study visits. Parent-incurred transportation, childcare, and out-of-pocket costs were relatively small and were reported only by a small percentage of parents who completed the 1-year follow-up survey. Only 9% of parents reported time for childcare for other children; the mean time required for childcare among them was 86 minutes per child.

**Table 1:** **Base case costs associated with the High Five for Kids intervention and usual care groups**, **micro**-**costing approach**

	**Usual care ($) (n = 192)**	**Mean cost/child ($)**	**Intervention ($) (n = 253)**	**Mean cost/child ($)**	**Range ($)**	**Mean time/child (minutes)**	**Range (minimum-maximum) (minutes)**
**Provider-incurred costs**							
Materials and equipment^a,b^	11,422	59	28,628	113	--	--	--
Study-related visits and phone counseling^c^	770	4	29,354	116	32 – 355 (Intervention)	100 (Intervention)	35-330 (Intervention)
					0-298	4	0-231
					(Usual Care)	(Usual Care)	(Usual Care)
**Parent-incurred costs**							
Parent Time^d^	--	--	6,014	24	0.00 – 85	65	0-240
Parent Transportation^e^	--	--	344	1	0.00 – 37	--	--
Parent Childcare^f^	--	--	451	2	0.00 – 50	8	0-210
Parent Out-of-pocket^g^	--	--	852	3	0.00 – 27	--	--
**TOTAL**	12,192	63	65,643	259			

^a^Intake forms, newsletters, printed materials, toys, scales, stadiometers.

^b^Non-standard equipment was used to get an accurate and precise measure of height and weight across sites. We did not account for the depreciation of equipment, as they were only used for two years.

^c^Cost of time APCs spent counseling patients. Includes wage ($44.05), fringe ($12.13), and overhead ($11.47 per visit) [21].

^d^Length of time parent spent at visit(s). Parent time valued at the U.S. Bureau of Labor Statistics 2007 mean U.S. adult wage converted to 2011 dollars ($21.87) [22].

^e^Amount of money parent spent to get to visit.

^f^Amount of time parent required childcare for other children during visit. Childcare cost was valued at the average household productivity wage rate ($13.28/hour) [19,24].

^**g**^Amount of out-of-pocket money parent spent on co-pays or other visit costs.

**Table 2:** **Base case net costs (with 95% confidence intervals)**^**a**^

	**Usual care**	**95% CI**	**Intervention**	**95% CI**
	**(n = 192)**		**(n = 253)**	
Costs ($)	12,192	[11,393, 13,174]	65,643	[64,522, 66,842]
Cost/child ($)	63	[59, 69]	259	[255, 264]
Net mean cost/child ($)			196	[191, 202]

^a^Accounts for provider-incurred costs and parent time costs detailed in Table 1.

### Sensitivity analyses

### Training costs

Training costs (Table 3) were a substantial component of non-medical costs and varied by staff discipline. Training costs for medical assistants in usual care practices who were responsible for measuring height and weight ranged from $16-24 per child. Medical assistants at each of the 10 intervention and usual care sites spent 1 hour in training each. APCs required substantially more training than other staff; training costs were $92-$176 per child. APCs spent a total of 254 hours in training in seven training sessions. Other clinical staff at the 5 intervention sites spent 2 hours in training each. The addition of staff training costs increased the cost estimates by 78%, or $152 per child, from the societal costing perspective. Cost estimates calculated using only APC training costs were still 44% higher than the base case results using the societal costing perspective.

**Table 3:** Sensitivity analyses on alternative costing approaches

	**Usual care ($) (n = 192)**	**Mean cost/child ($)**	**Range (minimum-maximum) ($)**	**Intervention ($) (n = 253)**	**Mean cost/child ($)**	**Range (minimum-maximum) ($)**
**Additional costs**						
Staff training^a^	4,570	24	--	44,489	176	--
APC training only^b^	2,993	16	--	23,316	92	--
**Health plan perspective costs**						
Reported study-related visits^c^	8,025	42	205 – 2,022	72,170	285	147 – 467
Reported intervention group study-related visits^d^	--	--	--	72,170	285	147 – 467
Total study-related visits^e^	8,025	42	205 – 2,022	162,255	641	147 – 467
Total intervention group study-related visits^e,d^	--	--	--	162,255	641	147 – 467
**Equipment costs**						
Annuitized equipment costs^f^	9,784	51	--	63,455	251	--

^a^Includes trainer salary costs, clinical staff time costs, and trainer and clinical staff travel costs. Clinical staff fringe was estimated at 30% of the hourly wage rate.

^b^Includes trainer salary costs, APC time costs, and trainer and APC travel costs.

^c^Based on provider reimbursements. 13 out of the 192 children in usual care had unexpected study-related weight visits. Reimbursement data was available for 238 study-related visits out of an estimated 504 study-related visits that occurred in the intervention group.

^d^Excludes unexpected usual care visit costs.

^e^Includes missing study-related visit costs that were not reported in provider reimbursement data, but that were imputed based on study visits attended.

^f^Equipment costs annuitized over 10 years at a discount rate of 3.5%.

### Health plan perspective costs

Costs reported by the health plan for study-related visits were $72,170 in the intervention group ($285 per child) and $8,025 in usual care ($42 per child). Expected intervention group health plan costs, estimated using imputation, were $162,255 total, or $641 per child. Costs estimated from the health plan perspective yielded cost estimates that were approximately 10% to 147% higher than base case results calculated using the societal costing perspective (Table 3).

### Equipment costs

In sensitivity analyses that considered two-year annuitized costs as opposed to the full cost of scales and stadiometers decreased costs in the intervention and usual care groups. Usual care costs decreased 19% from $63 to $51 per child. Intervention costs decreased only 3% from $259 to $251 per child (Table 3).

## Discussion

Intervention costs were higher than usual care costs in our base case and sensitivity analyses. From a societal perspective, the cost of High Five for Kids was $259 per child in the base case analysis compared to $63 per child in usual care, for a net cost of $196 per child (95% CI [$191, $202]). Provider-incurred costs were the primary driver of intervention costs. The cost of the intervention was higher in sensitivity analyses that considered the inclusion of training costs and costs calculated from the perspective of the health plan.

This analysis of the cost of High Five for Kids quantifies the economic burden of one primary care-based motivational interviewing intervention. Data on training costs and direct medical costs may be of particular interest to providers seeking to implement such an intervention, and information on costs from the perspective of the health plan may be of particular interest to payers. In order to get a better sense of the value of the intervention—the gains in health relative to the dollars spent on care—a cost-effectiveness analysis should be conducted. High Five for Kids measured program effectiveness in terms of changes in body mass index (BMI, kg/m^2^) and BMI z-score [4], but it is difficult to draw conclusions about the value of the program relative to other obesity interventions because reported effectiveness measures and time frames vary. A cost-effectiveness analysis could also translate biometric effectiveness measures into a more global health metric such as the quality-adjusted life year (QALY), which represents a summary measure of quality of life that includes all positive and negative aspects of a health state [19]. The use of QALYs would therefore allow cost-effectiveness analyses of obesity interventions to be directly compared to other childhood health interventions for obesity or other conditions.

Still, we can compare the cost of High Five to Kids to the cost of other pediatric obesity interventions. At $196 per child, the net cost of High Five for Kids falls within the range of four U.S. school- and community-based childhood obesity interventions, all calculated using a micro-costing approach and converted to 2011 dollars [20]. Planet Health cost $18 per child, the Coordinated Approach to Child Health (CATCH) intervention cost $119 per child, the FitKid afterschool intervention cost $427 per child, and the STORY intervention had a cost ranging from $594-$994 [5,12-14]. Several analyses have evaluated the cost of obesity interventions outside of the U.S.; the cost of these interventions ranges from less than a dollar to $2,183 per child [8,16,26,27] when calculated using similar categories of costs and adjusted for inflation and purchasing power parity [28].

Two phases of the Australian Live, Eat, and Play (LEAP) intervention, the only other primary care-based child obesity prevention program for which costs have been evaluated, were more costly than High Five for Kids. The net cost of delivering the LEAP intervention was approximately $852 (LEAP Phase 1) and $1,237 (LEAP Phase 2) per child from the health plan cost perspective. These estimates of the cost of LEAP include any reported costs of study-related visits with the primary care practitioner and equipment costs, but exclude any family-incurred time or out of pocket expenses [9-11,29]. Using similar categories of costs, the net cost of High Five for Kids ranged from $369-$725 per child. High Five for Kids was a more intensive intervention than LEAP, but LEAP was still more expensive than High Five for Kids, although some of the differences in costs may be attributable to differences in health care costs between countries and the use of nurse practitioners to conduct the intervention as opposed to physicians.

When making comparisons to other cost analyses, it is important to consider intervention characteristics or assumptions made in analyses that can affect per capita cost estimates. It is difficult to compare health plan perspective costs from other countries because the health services and unit costs may not be comparable [30]. Moreover, analysis methods such as the choice of cost perspective (government, health plan, or societal), measurement of cost offsets due to changes in healthcare utilization, or the time frame of the analysis can make cost estimates from some studies appear more favorable than others. For example, a 2009 Assessing Cost Effectiveness (ACE) study by Moodie et al. models a theoretical primary care intervention based on LEAP, applied to a greater population (n = 9,485) than the initial LEAP1 study (n = 82) [8]. A larger target population and the consideration of health care cost savings in adulthood from early weight loss results in a lower marginal cost per child for the ACE study than LEAP1. Given inconsistencies in methods and target populations, readers should be careful when interpreting and comparing the results of economic analyses, being sure to note the perspective, population size, and country of analysis, among other analysis methods and features of the intervention.

Our use of a micro-costing approach to calculate provider-incurred costs provides estimates of the marginal cost of the intervention per child treated, which may be useful to others planning programs. The micro-costing approach estimated substantially lower intervention costs than those generated using health plan perspective costs because health plan perspective costs may represent prices that could vary between practices and insurers, and may incorporate substantial administrative costs [31]. The micro-costing approach, which considers average wages and unit costs, may better reflect resource utilization.

We excluded training costs from our primary analysis, consistent with the conventional framework for economic evaluation that these were start-up fixed costs that would not vary materially with the scale of the intervention [32]. However, High Five for Kids APCs required repeated training sessions throughout the intervention time period that were not initially planned to refine their motivational counseling strategies. From that point of view, the costs of training APCs were not strictly start-up fixed costs and warranted examination in sensitivity analyses.

Because we were missing some health plan costs, we relied on imputation to derive expected costs from the health plan perspective. But because the children had a limited number of insurance providers and saw providers in one network, we believe that the imputed costs are fairly representative of actual costs. Secondly, the study was focused on a small number of practices in eastern Massachusetts, which may lead to concerns about the generalizability of study results to other practices in the U.S. However, the results found using the micro-costing approach represent national costs, improving generalizability of the findings. Lastly, the visit compliance among intervention subjects in primary care-based interventions such as High Five for Kids and LEAP was lower than expected and some usual care patients unexpectedly received weight counseling visits [11]. As the majority of intervention costs were comprised of visit costs, our results may underestimate the cost of an intervention with higher compliance.

## Conclusions

The net cost of the primary care-based High Five for Kids motivational interviewing intervention was $196 per child. In order to help decision makers decide whether to adopt such a program, future research should measure the value of High Five for Kids relative to other interventions using a cost-effectiveness analysis. A decision-analytic simulation model could explore scenarios in which the intervention would have been more or less effective in addition to illustrating the impact of the persistence of intervention effects beyond the trial. Estimating the short- and long-term value of the intervention using simulation models is also an important next step in appraising interventions.

## Abbreviations

CI: Confidence interval; BMI: Body mass index; APC: Advanced practice clinicians; LEAP: Live eat, and play intervention.

## Competing interests

The authors declare that they have no competing interests.

## Authors’ contributions

EMT, MWG, SLG, LAP, and DRW were responsible for the study concept and design. EMT, MWG, KHH, and CMH were responsible for the acquisition of data. DRW was responsible for the analysis and interpretation of data and drafted the manuscript. All authors reviewed the manuscript for intellectual content and approved the final manuscript.

## Pre-publication history

The pre-publication history for this paper can be accessed here:

http://www.biomedcentral.com/1472-6963/14/44/prepub
